# Writing centers, libraries, and medical and pharmacy schools

**DOI:** 10.5195/jmla.2020.714

**Published:** 2020-01-01

**Authors:** Melanie J. McGurr

**Affiliations:** Associate Professor of Bibliography and Head, Electronic Services, University of Akron, Akron, OH, mmcgurr1@uakron.edu

## Abstract

**Objective:**

This study investigated the existence of writing centers at medical and pharmacy schools, the location of those writing centers in a library or elsewhere, and librarians’ perceptions of how writing centers are viewed by students, faculty, and staff.

**Methods:**

A twelve-question survey was sent to libraries affiliated with a medical and pharmacy school in the United States.

**Results:**

Respondents were curious about writing centers, how they were viewed on campus, and how to start one. Overall, respondents described engagement with writing centers: 68% had a writing center on campus, 23% had a writing center in their library, and 11% had a writing center on the health sciences campus, including in the health sciences library. No respondents reported hearing negative comments from faculty or students about the writing centers, and 60% of respondents with writing centers that were available to medical and pharmacy students would recommend one to health sciences libraries without access to a writing center.

**Conclusion:**

This exploratory study showed that the establishment of writing centers in health sciences libraries is a topic of interest. Future studies could further investigate health sciences libraries’ roles in writing centers for pharmacy, medical, and other health sciences students.

## INTRODUCTION

A science-focused education usually has fewer writing or English requirements than a liberal arts degree. Yet, when students attend graduate programs at pharmacy and medical schools, there is an increasing emphasis on and expectation regarding writing, as well as presenting and publishing research. Pharmacy and medical students, including those who do not consider English their native language, may need specialized writing assistance. Some institutions offer support outside of the classroom by creating writing centers especially for medical and pharmacy students or collaborating with an already established writing center on campus to provide writing tutors for this student population.

For large medical or pharmacy schools associated with state or private universities that include undergraduate, graduate, and professional schools, finding writing assistance may be easier. Although medical and pharmacy schools are their own colleges within universities, students can take advantage of other support programs on campus, including writing centers. However, medical or pharmacy programs sometimes reside on their own campuses, making it more difficult for students to visit the “main” campuses. Furthermore, independent, stand-alone medical or pharmacy schools do not have access to services on other campuses, and they may have no dedicated writing support if they do not have a writing center on their campuses.

There is a small body of research on reflective and narrative writing in the medical, pharmacy, and nursing fields, but few articles exist on the use of writing centers or other writing support for medical or pharmacy students [1–7]. Writing center assistance for nursing students was described by White and Lamson, who formed a writing team and an online writing center to assist students [8]. Based on Latham and Ahern’s evaluation through focus groups and writing assessments of the writing support that nursing students need, faculty revised their courses and assignments and established a writing center [9]. McMillan and Raines discussed how a campus writing center is an important part of the suite of resources that nursing students need to succeed in their program, which also includes information literacy work with campus librarians and peer-review exercises with their fellow students [10].

The most extensive research on the subject of writing centers at a medical university are 2 articles about the Center for Academic Excellence and the Writing Center at the Medical University of South Carolina (MUSC) [11, 12], which is a stand-alone medical university with programs in dental medicine, graduate studies, health professions, medicine, nursing, and pharmacy. The MUSC Writing Center employs 4 full-time, tenure-track faculty as tutors. Writing center faculty visit classrooms, but most interactions take place one-on-one with students [11]. Through a 2-part method to gauge the “value and effectiveness of instruction by faculty with expertise in teaching writing” at the MUSC Writing Center [12], 1 in 4 students reported visiting the writing center at least once in the previous year, and 90% or more agreed that the writing center met their needs; the staff were competent, caring, and helpful; and the center hours and services were broad enough to help them. Also, students using the center were twice as likely to get an A on a target assignment.

At Northeastern Ohio Medical University (NEOMED), a public health sciences university and the author’s former institution, the investigation of writing center support for medical and pharmacy students arose when library staff began hearing from faculty that students were struggling with writing assignments and librarians began to notice the same problem. At this university, librarians are involved with a first-year interdisciplinary research course for medical and pharmacy students, including the grading of papers.

The library completed a brief pilot study of a writing center in the fall semester of 2015, which was funded by the library and approved by university administration. A science writing tutor with English as a second language (ESL) experience from a neighboring institution was hired. Promotional materials were created and distributed, faculty and student email discussion lists were used to publicize the pilot, information was added to the university learning management system, and the pilot was announced in certain first-year classes with a writing emphasis. The hours for the writing center were mostly in the late afternoon and evening to accommodate the schedules of medical and pharmacy students. The pilot was purposely held during the time of the largest writing assignment in the first-year curriculum. Although the writing center was not utilized much, students and faculty provided positive feedback and suggested that the writing center should continue to operate. The administration of the university asked for a written summary and analysis, which was provided, but no other action was taken.

This pilot study prompted a desire to further investigate writing centers for medical and pharmacy students. The goals of the present investigation were to assess how many universities offering medical or pharmacy programs in the United States had a writing center in the health sciences library, were planning to establish a writing center in the health sciences library, or offered writing help in other areas of campus; the types of writing services that were offered to medical and pharmacy students; and the perceived popularity of the writing center among students and faculty.

## METHODS

A survey instrument was created to explore how many US universities with medical and/or pharmacy programs had writing centers, how many were interested in establishing one, and how library administrators felt about writing centers on campus regardless of their actual presence. The survey was considered exempt by the Institutional Review Board of the University of Akron.

The survey consisted of twelve questions using split and display logic, so no respondent answered all twelve questions. Supplemental Appendix A provides the email sent with the survey instrument, and supplemental Appendix B provides the survey instrument. After ensuring that the respondents had a medical and/or pharmacy program at their university, the survey split along a simple line: Does your university have a writing center on campus?

In July 2017, the survey was sent to universities offering medical or pharmacy programs in the United States. Lists of universities offering a medical degree (MD) or doctor of osteopathic medicine (DO) were collected from the Association of American Medical Colleges and American Osteopathic Association, respectively [13, 14]. Universities offering advanced pharmacy degrees were collected from the American Association of Colleges of Pharmacy [15]. University websites were searched for confirmation of at least one of these programs and for information about the health sciences library on campus. If no specialized library existed, the main library was used for contact information.

The email address for the university librarian, dean, director, or head librarian was collected for all libraries serving medical and/or pharmacy students. An administrative email was chosen so that those schools without a separate web presence for a health sciences library were also included. An email to a dean or other administrator also meant that those schools with remote health sciences libraries would be reached. The author expected that the administrator would either complete the survey or ask another knowledgeable person in their organizations to provide the needed information. Universities that had both medical and pharmacy programs were checked for duplicates so that respondents would not receive two survey invitations. A few libraries did not have contact information available for any library personnel because of privacy walls or the use of “contact the library” forms. Because an email address could not be found, these libraries were not sent the survey.

A total of 230 emails were sent. Two non-deliverable messages and several out of office replies were received from the initial send. The 2 non-deliverable problems were resolved by sending the survey to other administrators at the institution. Reminder emails were sent at the 2-week and 3-week marks. Because the survey links were sent via email and not a survey tool, third and final reminder emails were sent in groups of 10 per email in case bulk emails were being caught by spam filters. A total of 144 responses were needed to achieve a 95% confidence level with a 5% confidence interval.

## RESULTS

A total of 56 responses were obtained; however, only 54 people completed the entire survey. This low response rate resulted in a 12% margin of error, which was higher than expected or desired.

Forty-one respondents had a College of Medicine, and 36 had a College of Pharmacy. A number of respondents chose the “Other” response to record additional health sciences offerings above and beyond medical and pharmacy at their university, such as schools of nursing, dentistry, health professions, biomedical sciences, public health, or other graduate programs.

When asked about the availability of a writing center, 68% of respondents stated they had a writing center on campus, 23% had a writing center in the library or available in the library, 34% had a writing center on the main campus, and 11% had a writing center on the health sciences campus but not in a library. One respondent noted that the writing center was located in the main library, but tutors visited the health sciences library upon request. Two respondents mentioned that their writing centers offered online help to medical and pharmacy students because the writing center was not on the same campus or not conveniently located for their students.

Depending on how respondents answered the question about writing center availability, they were then guided through one of the two survey tracks: the writing center question track or the non-writing center question track.

### Respondents with a writing center on campus

Of those universities with a writing center (n=38), assistance was offered with ESL (49%), science writing (e.g., lab reports and other work-related writing) (63%), and abstract and article writing in the sciences (52%). Other reported services were help with writing dissertations or theses, personal statements, citations, grant applications, reflective writing, and resumes and curricula vitae.

When asked if they had heard students commenting on the writing center, 60% of respondents reported hearing mostly positive comments, 34% had not heard comments, 6% heard mostly neutral comments, and no one heard mostly negative comments. When asked if they had heard faculty or staff commenting on the writing center, 49% of respondents reported hearing mostly positive comments, 39% had not heard comments, 12% heard mostly neutral comments, and no one heard mostly negative comments.

Most respondents (60%) stated that they would recommend a writing center to other medical or pharmacy schools, 3% would not recommend a writing center to other medical or pharmacy schools, and 37% were not sure.

### Respondents without a writing center on campus

Respondents from universities without a writing center (n=16 complete survey responses) were asked if students had access to a tutor or writing expert on campus. Most respondents (56%) answered “yes,” 38% answered “no,” and 6% responded “other.” One respondent clarified the “other” field by commenting that a librarian checked formatting for theses and dissertations only.

When asked if they had heard students suggest or discuss a writing center, 50% of respondents answered “yes,” 38% answered “no,” and 12% answered “maybe.” When asked if they had heard faculty or staff suggest or discuss a writing center, 56% answered “yes,” 38% answered “no,” and 6% answered “maybe.” Overall, 69% of respondents thought a writing center would benefit their medical or pharmacy campus, 12% did not think a writing center would benefit their medical or pharmacy campus, and 19% were not sure. One respondent mentioned that it was on their agenda to discuss with their boss; another said that medical students at their university do not typically have writing assignments; and one mentioned that students needed help, but this need would not necessarily be met by another service unit.

### All respondents

All respondents were asked if there was anything they would like to add about writing centers for medical or pharmacy students. Twenty people responded with comments including questions asking how to get a writing center started, comments on how the writing center started on their campus, and opinions about what the students expected or wanted versus the reality of a writing center.

Throughout the survey, nine respondents mentioned in the comments the library or medical school as a satellite or main writing center location. Some respondents explained that the main campus writing center sent a tutor to the medical, pharmacy, or satellite campus on a set schedule. One respondent mentioned a writing center on the main campus but was unsure if it could offer the type of support that medical and pharmacy students needed. Five respondents noted that they were in varying stages of discussing or planning a writing center on their health sciences campuses. Two respondents specifically emailed the author asking for data from the survey in case it would be helpful to their efforts to create a writing center for health sciences students in their libraries or on their campuses. One comment mentioned that it required the Higher Learning Commission to get the writing center to have a presence on their health sciences campus, and the service was now heavily used. One commenter said that a faculty member arranged to have a tutor come to the satellite campus from the main campus.

## DISCUSSION

Although the survey response rate was low, the information gathered serves as an observatory exercise to start a conversation about the importance of writing centers for pharmacy and medical students in the United States and roles for health sciences libraries and librarians. Future studies could solicit respondents through direct emails, phone calls, or email discussion lists rather than sending survey invitations to library deans or directors.

Of those respondents who did not have a writing center on their health sciences campuses, 69% were interested in one. Research on writing centers for health sciences students mentions partnering with faculty, academic support services, and even community-based groups [8, 9]. A few respondents mentioned faculty members from the English department or staff from Student Affairs as being responsible for starting or supporting a writing center on campus. A number of commenters mentioned collaborating with other departments on campus and taking advantage of any writing assistance that was already available to their institutions. White and Lamson’s results show that their students used the online components of the writing center more than the face-to-face resources, so planning for an online presence may be wise, regardless [8]. If a tutor cannot actually visit the medical and pharmacy campus, perhaps they can hold virtual office hours, provide videos, or provide other online support.

There are many avenues for future research on this topic, including investigations into how writing centers are funded and how they function administratively in a library. Further investigations should also include how to organize and start writing centers for pharmacy and medical students, especially in regard to recruiting tutors and securing funding. In addition, assessing the effectiveness of face-to-face versus online assistance and the range of services offered by writing centers could help universities make informed decisions about how to better support student writing.

## SUPPLEMENTAL FILES

Appendix A Script for email

Appendix B Survey instrument

## 

**Melanie J. McGurr**, mmcgurr1@uakron.edu, https://orcid.org/0000-0001-6120-147X, Associate Professor of Bibliography and Head, Electronic Services, University of Akron, Akron, OH

## References

[1] Clemmons AB, Hoge SC, Cribb A, Manasco KB (2015). Development and implementation of a writing program to improve resident authorship rates. Am J Health Syst Pharm.

[2] Cowen VS, Kaufman D, Schoenherr L (2016). A review of creative and expressive writing as a pedagogical tool in medical education. Med Educ.

[3] Kerr L (2010). More than words: applying the discipline of literary creative writing to the practice of reflective writing in health care education. J Med Humanit.

[4] King AE, Joseph AS, Umland EM (2017). Student perceptions of the impact and value of incorporation of reflective writing across a pharmacy curriculum. Curr Pharm Teach Learn.

[5] Rawson RE, Quinlan KM, Cooper BJ, Fewtrell C, Matlow JR (2005). Writing-skills development in the health professions. Teach Learn Med.

[6] Wear D, Zarconi J, Garden R, Jones T (2012). Reflection in/and writing: pedagogy and practice in medical education. Acad Med.

[7] Yanoff KL, Burg FD (1988). Types of medical writing and teaching of writing in U.S. medical schools. J Med Educ.

[8] White BJ, Lamson KS (2017). The evolution of a writing program. J Nurs Educ.

[9] Latham CL, Ahern N (2013). Professional writing in nursing education: creating an academic-community writing center. J Nurs Educ.

[10] McMillan LR, Raines K (2011). Using the “write” resources: nursing student evaluation of an interdisciplinary collaboration using a professional writing assignment. J Nurs Educ.

[11] Smith TG, Ariail J, Richards-Slaughter S, Kerr L (2011). Teaching professional writing in an academic health sciences center: the writing center model at the Medical University of South Carolina. Teach Learn Med.

[12] Ariail J, Thomas S, Smith T, Kerr L, Richards-Slaughter S, Shaw D (2013). The value of a writing center at a medical university. Teach Learn Med.

[13] Association of American Medical Colleges (2017). AAMC medical school members [Internet].

[14] American Osteopathic Association (2017). Osteopathic medical schools [Internet].

[15] American Association of Colleges of Pharmacy (2017). AACP institutional membership [Internet].

