# Supplementary material for: Writing centers, libraries, and medical and pharmacy schools
Source: J Med Libr Assoc. 2020 Jan 1;108(1):84–8. doi: 10.5195/jmla.2020.714 (PMC6919988; doi:10.5195/jmla.2020.714)
Supplement: Appendix A [file jmla-108-84-s001.pdf]

JMLA Journal of the Medical Library Association

SC

Supplemental content to
J Med Libr Assoc. Jan;108(1):dx.doi.org/10.5195/jmla.2020.714
www.jmla.mlanet.org
© McGurr 2020

Writing centers, libraries, and medical and pharmacy schools

Melanie J. McGurr

APPENDIX A

Script for email

The following survey asks questions concerning whether writing centers (WCs) are available to medical or pharmacy students on campus. The increased emphasis on reflective and research writing in the pharmacy and medical curriculum, along with the expectation of presenting and publishing research, may cause some students to struggle with this aspect of their education. Many institutions are offering support outside of the classroom, either creating a new WC or collaborating with a larger WC on campus. These survey questions explore how many medical and pharmacy schools have a WC or writing tutors available to their students, how many are located in libraries, and how many colleges are interested in adding this service for students if current support is not in place.

This survey is being sent to the director or dean at health sciences or main campus libraries because libraries, or learning centers, are often the site of a WC, WC satellites, or writing tutor space on campus. Using targeted email addresses helps to keep one response per institution, but if you are not the best fit to respond to the survey, please forward the survey to the appropriate person.

The survey is an Internet survey that is twelve questions long and should take approximately ten minutes to complete. No responses are required, and your participation is voluntary. You can quit the survey at any time, and your answers are anonymous and confidential, and no demographic information is asked. The survey tool will collect Internet protocol (IP) information, but that information will not be collected for analysis or discussion. Data will be reported in aggregate forms for any presentations or publications.

Questions or concerns about this study can be directed to mmcgurr1@uakron.edu. Any questions about your rights as a participant or the study can be directed to the University of Akron Institutional Review Board (330.972.7666).
