# Supplementary material for: Writing centers, libraries, and medical and pharmacy schools
Source: J Med Libr Assoc. 2020 Jan 1;108(1):84–8. doi: 10.5195/jmla.2020.714 (PMC6919988; doi:10.5195/jmla.2020.714)
Supplement: Appendix B [file jmla-108-84-s002.pdf]

Journal of the Medical Library Association

Supplemental content to
J Med Libr Assoc. Jan;108(1):dx.doi.org/10.5195/jmla.2020.714
www.jmla.mlanet.org
© McGurr 2020

Writing centers, libraries, and medical and pharmacy schools

Melanie J. McGurr

APPENDIX B

Survey instrument

Q1. Does your university have a…
- College of Medicine (1)
- College of Pharmacy (2)
- Other (3) ________________

Q2. Does your university have a writing center or writing lab on campus?
- Yes (1)
- No (2)

Q3. If yes, the writing center is…
- In the library or services are available in the library (1)
- On main campus (2)
- Not in the library, but on the health sciences campus (3)
- Other (4) ________________

Q4. Does the writing center offer assistance with…
- English as a second language (1)
- Science writing (2)
- Abstract or article writing in the sciences (3)
- I'm not sure (4)
- Other (5) ________________

Q5. If you do have a writing center, have you heard students comment on it?
- Yes, in a mostly positive way (1)
- Yes, in a mostly negative way (2)
- Yes, in a mostly neutral way (3)
- I have not heard any comments (4)

Q6. If you do have a writing center, have you heard faculty or staff comment on it?
- Yes, in a mostly positive way (1)
- Yes, in a mostly negative way (2)
- Yes, in a mostly neutral way (3)
- I have not heard any comments (4)

Q7. Would you recommend it to other medical or pharmacy schools?
- Yes (1)
- No (2)
- Not sure (3)

JMLA Journal of the Medical Library Association

SC

Supplemental content to
J Med Libr Assoc. Jan;108(1):dx.doi.org/10.5195/jmla.2020.714
www.jmla.mlanet.org
© McGurr 2020

Q8. If no, do the students have access to a tutor or writing expert on campus?
- Yes (1)
- No (2)
- Not sure (3)

Q9. If you don't have a writing center or any writing help for medical or pharmacy students, have you heard students suggesting or discussing a writing center?
- Yes (1)
- No (2)
- Not sure (3)

Q10. If you don't have a writing center or any writing help for medical or pharmacy students, have you heard faculty or staff suggesting or discussing a writing center?
- Yes (1)
- No (2)
- Not sure (3)

Q11. Is a writing center something you think would benefit your medical or pharmacy campus?
- Yes (1)
- No (2)
- Not sure (3)

Q12. Is there anything else you would like to add about writing centers for medical or pharmacy students?

____
